# Oxysterol-Binding Protein 2 Promotes Pancreatic Ductal Adenocarcinoma Progression Through Epithelial-Mesenchymal Transition

**DOI:** 10.3389/fonc.2021.762233

**Published:** 2022-01-20

**Authors:** Shuai Huang, Xudong Zhang, Kai Luo, Li Jiang, Jianhua Jiang, Renfeng Li

**Affiliations:** ^1^ Departments of Hepatobiliary and Pancreatic Surgery, The First Affiliated Hospital of Zhengzhou University, Zhengzhou, China; ^2^ Department of Radiotherapy, The First Affiliated Hospital of Zhengzhou University, Zhengzhou, China; ^3^ Department of General Surgery, Hua County People’s Hospital, Anyang, China

**Keywords:** epithelial–mesenchymal transition, oxysterol-binding protein 2, pancreatic ductal adenocarcinoma, migration, invasion, proliferation, apoptosis

## Abstract

Oxysterol-binding protein 2 (OSBP2) is crucial for promoting the growth and development of cancers; however, its effects on pancreatic ductal adenocarcinoma (PDAC) are still unclear. Here, we report that OSBP2 is an efficient tumor-associated protein to lead to extremely malignant characteristics in PDAC. We discovered that increased OSBP2 expression in primary tumors was associated with shorter survival in PDAC patients. Therefore, we used immunohistochemistry (IHC) to analyze the levels of OSBP2 expression in PDAC tissues and adjacent paracancerous tissues. We used wound healing and Transwell assays to evaluate the effects of OSBP2 on PDAC cell (ASPC-1 and BXPC-3) migration and invasion, respectively, and CCK-8 and Annexin V/PI double staining to evaluate the effects of OSBP2 on PDAC cell proliferation and apoptosis, respectively. Western blotting was used to analyze the effect of OSBP2 on the PDAC cell phenotype. We also explored the effect of OSBP2 on chemosensitivity to gemcitabine (GEM) and 5-fluorouracil (5-FU). We validated these findings in an *in vivo* mouse model. The data show that OSBP2 overexpression promoted PDAC cell migration, invasion, proliferation and chemotherapy resistance, and decreased apoptosis. OSBP2 overexpression downregulated E-cadherin expression and upregulated N-cadherin, vimentin, Snail, Slug, ZEB1, and β-catenin expression. Taken together, our findings indicated that OSBP2 was overexpressed in PDAC and that upregulation of OSBP2 may promote PDAC progression. Therefore, OSBP2 may have potential diagnostic and therapeutic value in PDAC.

## Introduction

Pancreatic ductal adenocarcinoma (PDAC) is a serious health problem worldwide owing to its poor prognosis, five-year survival rate of less than 5%, and ranks as the eighth most common cause of cancer-related deaths globally (1–3). In China, the median survival of PDAC patients is 7.8 months, with 30.0% of patients undergoing effective radical resection and 9.8% of patients undergoing synthetic curative treatment (4, 5). However, the causes of PDAC remain unclear. Although tremendous efforts have been made and there has been great progress in this field over the last few decades, PDAC patient survival has not exhibited obvious improvement (6, 7). Its aggressive biological phenotype that elicits early local invasion and metastasis features causes low survival rates (8, 9). Therefore, we need to further elucidate the molecular mechanisms behind the occurrence, development, treatment resistance and metastasis of this deadly disease.

Cancer cell invasion and metastasis are very complex processes that involve many factors, and the extracellular matrix plays essential roles in the initiation and maintenance of tumor metastasis (10–12). The most critical relevant mechanism mediating the metastatic cascade is the epithelial–mesenchymal transition (EMT) process in cancer cells, which is also a primary step in the induction of tumor cell migration and invasion (13, 14). EMT plays important roles in embryonic development and the differentiation of multiple tissues and organs and also in the metastasis of cancer cells (15–17). This process is characterized by a loss of cell-to-cell or extracellular matrix adhesions *via* inhibition of epithelial markers, namely, E-cadherin, and acquisition of mesenchymal features, such as upregulated expression of the mesenchymal markers N-cadherin and vimentin (18, 19). Cells undergoing EMT are endowed with increased migratory and invasive properties (20), and a large number of studies have demonstrated that EMT is involved in the development and progression of PDAC (21–23).

The oxysterol-binding protein (OSBP) and OSBP-related protein (ORP) family comprises twelve mammalian genes, and its members are characterized by a conserved OSBP homology domain (OHD) that binds sterols and lipids, a pleckstrin homology (PH) domain and two phenylalanine residues in an acidic tract (FFAT) motif that mediates interactions with the membranes of organelles (24). A great number of studies have demonstrated that some OSBP/ORP members participate in cancer progression, but how OSBP/ORP family members influence cancer cell initiation and progression and the mechanism behind these processes remain unclear. OSBP2, which is also named ORP4, is expressed in the human brain, heart, and testis but not in other human tissues or mouse tissues (25, 26). ORP4 plays an important role in human malignant tumor cell proliferation and survival (27) and is a target of the natural antiproliferative steroidal saponin OSW-1 (28), and published studies show that OSBP2 plays a critical role in the control of oncogenic cell growth. At present, no evidence has identified a relationship between PDAC and OSBP2.

This study is the first to demonstrate that OSBP2 may work as a tumor-associated protein that significantly affects the malignant behaviors of PDAC. These behaviors could be significantly enhanced when OSBP2 was overexpressed, while downregulation of OSBP2 decreased the migration and invasion abilities of PDAC cells *in vitro* and *in vivo*. Additionally, we showed that OSBP2 was involved in the EMT process of PDAC.

## Materials and Methods

### Cell Culture and Reagents

We purchased the human PDAC cell lines ASPC-1 and BXPC-3 from American Type Culture Collection (ATCC) and cultured them in complete growth medium according to the manufacturer’s instructions. Cells were maintained in an incubator at 37°C and 5% CO_2_ and tested for mycoplasma every 3 months (last verified negative date, May 1, 2020). The experiments in this study used cells within ten passages after thawing.

The following reagents were used. RPMI 1640 cell culture medium was purchased from Gibco. Fetal bovine serum (FBS) was obtained from Biological Industries. Pancreatic enzyme and EDTA were acquired from Sigma. Phosphate-buffered saline (PBS) was from DHeLix.

### Tissue Specimens

We obtained 100 PDAC specimens from patients who underwent pancreatic surgical resection and provided informed consent before undergoing surgery. None of the patients received radiotherapy or chemotherapy before surgery. The resected specimens were immediately frozen at −80°C, after which tumor and adjacent nontumor tissues were sampled (approximately 1 cm^3^ of each) and pathologically examined to confirm the patient diagnosis. This study was approved by the Ethics Committee of The First Affiliated Hospital of Zhengzhou University and authorized by Zhengzhou University.

### OSBP2 Knockdown and Overexpression in PDAC Cells

We cloned full-length human OSBP2 cDNA into the pcDNA3.1 plasmid (Invitrogen Co., Ltd. China) to induce stable OSBP2 expression, and Jima Genomics Co., Ltd. designed the siRNAs targeting OSBP2 (siRNA-OSBP2) to knock down OSBP2 expression. In short, cells were seeded at a density of 5 × 10^5^ cells/well in 6-well plates in medium containing 10% FBS. When the cells reached 80% confluency, they were transfected with 50 nmol/L siRNAs in Lipofectamine 3000 (Invitrogen Co., Ltd. China) for 48 h. Stably transfected cells were selected by Puromycin for 7–14 days, and we used western blotting to evaluate the level of OSBP2 expression. Details are presented in **Figure 8**. The experiment was repeated three times.

### Mouse Strains

We obtained female BALB/c nude mice and NOD/SCID mice from the Laboratory Animal Centre of Zhengzhou University. Procedures relating to the care and use of mice in this study were conducted in accordance with the National Institutes of Health (NIH) guidelines, with the animals housed in an accredited laboratory animal care facility. All animal studies were carried out in accordance with approved Institutional Care and Use Committee protocols at Zhengzhou University.

### *In Vivo* Subcutaneous Injection

Female BALB/c nude mice aged 4 to 6 weeks were maintained in a barrier facility on high-efficiency particulate air-filtered racks. Twenty mice were divided into four groups (5 mice/group) that were injected with the following cell types (10^7^ cells/injection): OSBP2-knockdown ASPC-1 cells, control ASPC-1 cells, OSBP2-overexpressing BXPC-3 cells, and control BXPC-3 cells. The cells were subcutaneously injected into the right posterior back of the nude mice. Tumor nodules were observed weekly, and the length of the tumor was measured. Four weeks later, all tumor nodules were collected and fixed with 4% paraformaldehyde for subsequent experiments.

### *In Vivo* Caudal Vein Injection

Female NOD/SCID mice aged 3 to 4 weeks were maintained in a barrier facility on high-efficiency particulate air-filtered racks. Twenty mice were divided into four groups (5 mice/group) that were injected with the following cell types (10^7^ cells/injection): OSBP2-knockdown ASPC-1 cells, control ASPC-1 cells, OSBP2-overexpressing BXPC-3 cells and control BXPC-3 cells. The cells were injected *via* tail vein and observed for 8 weeks. Eight weeks later, all of the mice were sacrificed, lung tissues were removed, and the wet weight of both lungs was measured before they were fixed with 4% paraformaldehyde for subsequent experiments.

### Western Blot

We extracted total proteins from cells and tissue by using RIPA lysis buffer with 1% phenylmethanesulfonyl fluoride and quantified the protein concentration using a bicinchoninic acid protein assay kit (Wanleibio Co., Ltd., China). We used sodium dodecyl sulfate-polyacrylamide gel electrophoresis (SDS-PAGE) to denature and separate 20 µg of protein per sample. Then, the proteins were transferred onto polyvinylidene difluoride membranes (EMD Millipore, Billerica, MA, USA), which were blocked in 5% skim milk powder (Yili Co., Ltd., China) in Tris-buffered saline-Tween-20 (TBS-T). Then, we incubated the membranes with the corresponding primary antibody overnight at 4°C, rinsed them 4 times with TBS-T for 5 min per wash, and incubated them with horseradish peroxidase-conjugated secondary antibody for 45 min at 37°C. The secondary antibody is goat anti−rabbit IgG−HRP anti-bodies (Wanleibio Co., Ltd., China). The membranes were treated with an enhanced chemiluminescence (ECL) reagent (EMD Millipore) to develop the protein bands. Antibodies targeting the proteins E-cadherin (cat. no. 3195), N-cadherin (cat. no. 13116), Vimentin (cat. no. 5741), Snail (cat. no. 3897), β-catenin (cat. no. 8480), Slug (cat. no. 9585), and ZEB1 (cat. no. 3396) were purchased from Cell Signaling Technology, Inc. (Danvers, MA, USA). Antibody targeting β-actin was purchased from Wanleibio. All antibodies were used in accordance with the manufacturer’s instructions. The primary antibodies were diluted at a ratio of 1:1,000, and the secondary antibodies were diluted at a ratio of 1:5,000 according to the manufacturer’s instructions.

### Proliferation Assay

Different cell lines (ASPC-1, OSBP2-knockdown ASPC-1; BXPC-3, OSBP2-overexpressing BXPC-3) were cultured until they reached approximately 90% confluence, after which they were washed once with PBS (DHeLix Co., Ltd., China). Then, we added an appropriate volume of 0.25% trypsin (Sigma Co., Ltd., USA) to disrupt the cell from the culture plates, and when the cells became round, complete medium was added to terminate the reaction. Next, we agitated the cells in the cell culture plate with a 5 ml pipettor, collected the suspension into a 15 ml test tube, and centrifuged the suspension at 88*g* for 3 min. The supernatant was removed, 1 ml of complete medium was added, and the cells were counted. Cells were seeded into 96-well plates (5 × 10^3^ cells/well) according to the experimental groups. Then, the CCK-8 test was performed after cells were cultured in an incubator at 37°C with 5% CO_2_ for 0, 24, 48, and 72 h. At the indicated times, the supernatant was discarded, 100 μl of complete medium and 10 μl of CCK-8 were added to each well, and the cells were cultured in an incubator at 37°C with 5% CO_2_ for an additional 2 h. The optical density (OD) value at 450 nm in each well was measured on a standard microplate reader (BIOTEK Co., Ltd., USA).

### Cytotoxicity Assay

Cells were seeded at low density (5,000 cells per well) in 96-well plates and treated with different concentrations (0–2,000 μmol/l) of 5-fluorouracil (5-FU) or gemcitabine (GEM). After 72 h of incubation, we used the CCK-8 test to measure the OD value at 450 nm, as mentioned above.

### Transwell Assay

We used Transwell chambers (Corning Inc. USA) to evaluate cell migration and invasion. Matrigel (Corning Inc. USA) was applied to the upper surface of the membranes in the invasion assay. Cells suspended in serum-free medium were added to the upper chamber of the Transwell inserts (6 × 10^3^ cells), and 800 μl of medium with 30% FBS was added to the lower chamber. After incubation, the Transwell membranes were washed twice with PBS, fixed with 4% paraformaldehyde at room temperature for 20 min, stained with 0.5% crystal violet solution for 5 min, and rinsed with distilled water. The cells that migrated or invaded to the lower chamber were counted under an inverted microscope (Motic Electric Group Co., Ltd., Xiamen, China) at 200× magnification.

### Wound Healing Assay

Different cell lines (ASPC-1 cells, OSBP2-knockdown ASPC-1 cells, BXPC-3 cells, OSBP2-overexpressing BXPC-3 cells) were cultured until they reached 100% confluence. Then, the medium was replaced with serum-free medium, and 1 μg/ml mitomycin C was added to the cells for 1 h, after which the monolayers were scratched with a 200 μl pipette tip, washed with serum-free medium to remove cellular debris, observed under a microscope and photographed the positions of the cells in the photos were noted in preparation for subsequent photos. The scratched cultures were incubated in serum-free medium in an incubator at 37°C and 5% CO_2_ for 48 h and then photographed again.

### Apoptosis Assay

The Annexin V-FITC/PI Apoptosis Detection kit (BD Biosciences, San Jose, CA, USA) was used to assess apoptosis according to the manufacturer’s instructions. In brief, we washed cells in ice-cold PBS and applied Annexin V-FITC and PI solutions to the cells in the dark for 15 min. Next, we used a FACScan flow cytometer to measure apoptosis rates. Finally, we summarized the data as the mean ± standard error (SD).

### Immunohistochemistry (IHC)

Fixed tissues were sliced into 5 µm-thick sections. After they were heated to 60°C for 2 h, the specimens were deparaffinized using xylene at room temperature and rehydrated in a descending series of ethanol (95, 85, and 75%). For IHC staining, endogenous peroxidase activity was blocked by treatment with 3% hydrogen peroxide for 15 min. Tissues were then stained with primary antibodies for 12 h at 4°C according to the manufacturer’s instructions followed by staining with secondary antibodies. The processed sections were observed under a microscope (OLYMPUS Co., Ltd., Japan) at a magnification of 400× and imaged.

### Hematoxylin and Eosin (H&E) Staining

Fixed tissues were sliced into 5 µm-thick sections. After they were heated to 60°C for 1 h, the specimens were deparaffinized using xylene at room temperature and rehydrated in a descending series of ethanol (95, 85, and 75%). Subsequently, the samples were stained with hematoxylin for 5 min and eosin for 3 min at room temperature. Then, after dehydration, the sections were mounted, viewed under a microscope (200×; OLYMPUS Co., Ltd., Japan) and imaged.

### Microarray Analysis

PDAC tissues and adjacent paracancerous tissues for the tissue microarray (TMA) were obtained at the First Affiliated Hospital of Zhengzhou University. First, an experienced pathologist selected the 1 mm-core area; then, we used a TMA Grand Master (3DHISTECH) to drill and place the recipient blocks. Three cores were obtained from each primary tumor, and 1–3 cores were obtained from the metastatic lymph nodes. We coded and randomly placed the cores on the array. Relevant clinical information was extracted from hospital cases. To compare staining intensity and survival, at least two cores with histological scores are required per patient. In the process of tissue staining analysis, the pathologists were blinded to the clinical data.

### Statistical Analysis

We used SPSS 16.0 (IBM) to conduct statistical analysis in this study. The data are presented as the means ± SD. All *in vitro* experiments were conducted independently in triplicate. Comparisons between treatment groups were performed by Student’s t-test. Statistical analysis was conducted with either Microsoft Excel or Origin Labs. The Kaplan–Meier method was used to compare overall survival (OS) among patients in different groups, and the log-rank test was used to estimate differences in survival. Univariate and multivariate analyses were based on the Cox proportional hazards regression model. P <0.05 was considered statistically significant.

## Results

### Overexpression of OSBP2 is Correlated With Shorter Survival in PDAC

**Figure 1:**
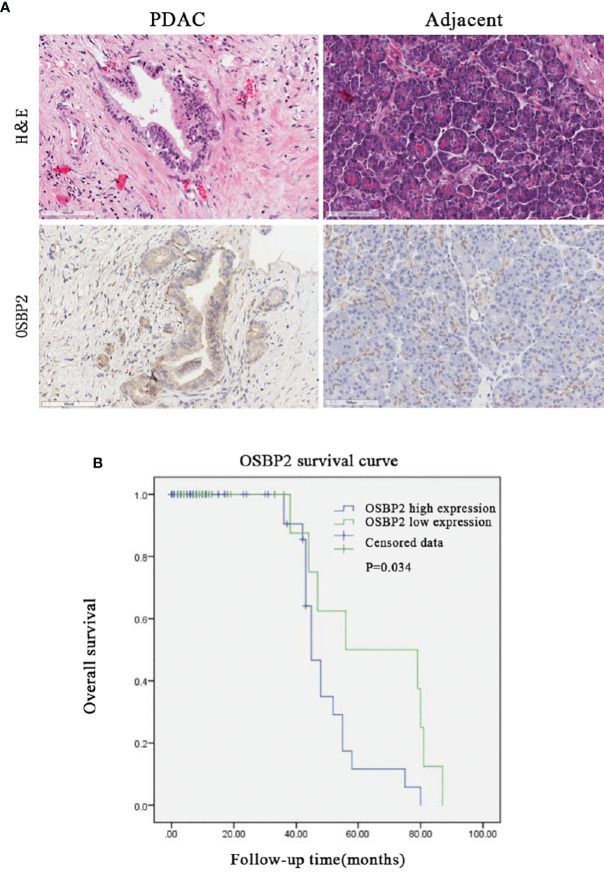
High OSBP2 expression in PDAC correlates with shorter survival. **(A)** Expression of OSBP2 in pancreatic cancer tissues and paired adjacent paracancerous tissues (Scale bars, 100 μm). **(B)** Association between OSBP2 expression in tumors and overall survival in 100 PDAC patients (P = 0.034 by log-rank test). OSBP2, oxysterol-binding protein 2; PDAC, pancreatic ductal adenocarcinoma.

The relationship between the clinicopathological characteristics of PDAC patients and OSBP2 expression is revealed in **Table 1**. Our data demonstrated that OSBP2 expression in PDAC tissues were related to nerve invasion (*P <0.05*) and pathological grading (*P <0.05*) but were not related to age, sex, lymph node metastasis or AJCC stage. The IHC staining results showed that OSBP2 expression was higher in PDAC tissues than in adjacent paracancerous tissues (**Figure 1A**). We used Kaplan–Meier curves to analyze the relationship between OSBP2 expression in PDAC and OS of patients. Our data revealed that high OSBP2 expression was related to shorter survival of these patients (**Figure 1B**). Therefore, we further investigated the effect of OSBP2 expression on the biological behavior of PDAC cells.

**Table 1:** Relationship between the expression level of OSbp2 and the clinicopathological parameters of pancreatic cancer patients.

Clinical parameters	N	OSBP2 expression		p-value
		High	Low	
		[n (%)]	[n (%)]	
Sex				0.284
Male	63	49 (77.8)	14 (22.2)	
Female	37	32 (86.5)	5 (13.5)	
Age (years)				0.135
<60	47	41 (87.2)	6 (12.8)	
≥60	53	40 (75.5)	13 (24.5)	
Nerve invasion				0.011
Yes	58	52 (89.7)	6 (10.3)	
No	42	29 (69.0)	13 (31.0)	
Pathological grading				0.018
I	12	6 (50.0)	6 (50.0)	
II	78	66 (84.6)	12 (15.4)	
III	10	9 (90.0)	1 (10.0)	
AJCC stage				1.000
I-II	98	79 (80.6)	19 (19.4)	
III–IV	2	2 (100)	0 (0)	
Lymph node metastasis				0.427
Absent (N0)	55	43 (78.2)	12 (21.8)	
Present (N1–3)	45	38 (84.4)	7 (15,6)	

All data are expressed as the number of patients (%). P-values were calculated using SPSS 17.0 using a Chi-square test. P-values <0.05 were considered to indicate statistical significance. OSBP2, Oxysterol-binding protein 2; AJCC stage, American cancer Joint Committee stage.

### OSBP2 Promotes the Migration and Invasion of PDAC Cells

**Figure 2:**
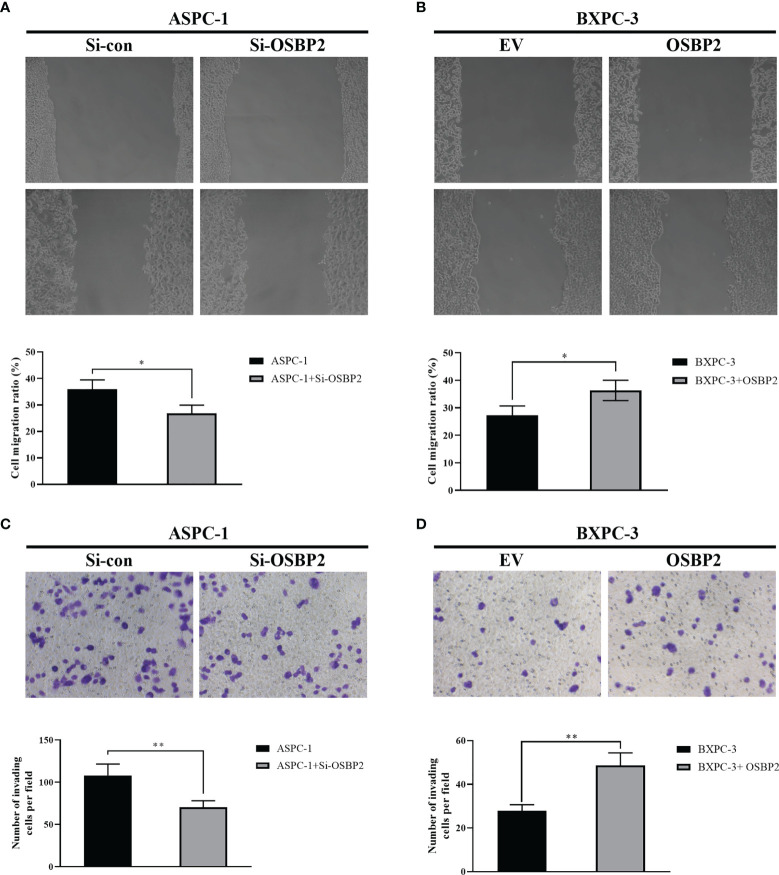
Effect of OSBP2 on PDAC cell migration and invasion. **(A)** Low OSBP2 expression inhibited ASPC-1 cell migration (P <0.05). **(B)** Overexpression of OSBP2 promoted BXPC-3 cell migration (P < 0.05). **(C)** Low OSBP2 expression inhibited ASPC-1 cell invasion (P <0.01). **(D)** Overexpression of OSBP2 promoted BXPC-3 cell invasion (P <0.01). OSBP2, oxysterol-binding protein 2; PDAC, pancreatic ductal adenocarcinoma; Si-OSBP2, siRNA targeting OSBP2; EV, empty vector. *P < 0.05, **P < 0.01.

We studied the role of OSBP2 in PDAC cell mobility by inhibiting OSBP2 expression in ASPC-1 cells using a specific siRNA and performing a wound healing assay. The results revealed that OSBP2 knockdown reduced wound closure compared with that of the control ASPC-1 cells (*P <0.05*; **Figure 2A**). We next overexpressed OSBP2 in BXPC-3 cells and performed the wound healing assay, which revealed that OSBP2 overexpression led to increased cell migration compared with empty vector-treated BXPC-3 cells (*P <0.05*; **Figure 2B**). Transwell assays were employed to further evaluate the impact of OSBP2 on PDAC cell invasion, which showed that PDAC cell invasion was suppressed by OSBP2 inhibition in ASPC-1 cells (*P <0.01*; **Figure 2C**) and increased by OSBP2 overexpression in BXPC-3 cells (*P <0.01*; **Figure 2D**).

### OSBP2 Decreases the Apoptosis of PDAC Cells

**Figure 3:**
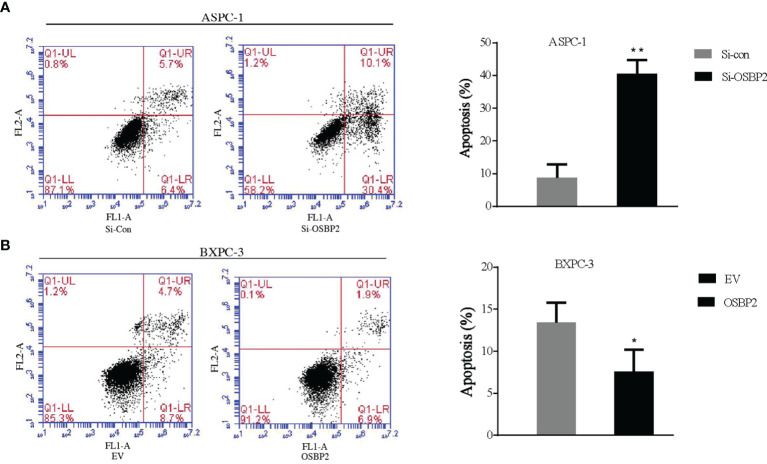
Effect of OSBP2 on PDAC cell apoptosis. **(A)** Low OSBP2 expression increased tumor cell apoptosis in the ASPC-1 cell line. **(B)** Overexpression of OSBP2 decreased tumor cell apoptosis in the BXPC-3 cell line. OSBP2, oxysterol-binding protein 2; PDAC, pancreatic ductal adenocarcinoma; Si-OSBP2, siRNA targeting OSBP2; EV, empty vector. *P < 0.05 **P < 0.01.

We investigated the effect of OSBP2 on PDAC cell apoptosis. OSBP2 inhibition significantly increased the number of apoptotic cells and decreased the number of tumor cells compared with those of the control ASPC-1 cells (**Figure 3A**). By contrast, OSBP2 overexpression dramatically decreased the number of apoptotic cells and increased the number of tumor cells compared with those of empty vector-treated BXPC-3 cells (**Figure 3B**).

### OSBP2 Promotes the Proliferation of PDAC Cells

**Figure 4:**
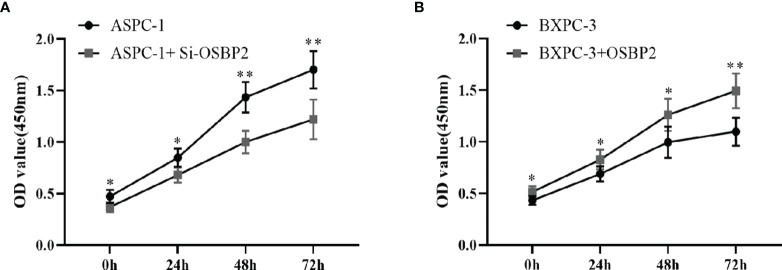
Effect of OSBP2 on PDAC cell proliferation. **(A)** Low OSBP2 expression decreased tumor cell proliferation in the ASPC-1 cell line (P < 0.01). **(B)** Overexpression of OSBP2 increased tumor cell proliferation in the BXPC-3 cell line (P < 0.01). OSBP2, oxysterol-binding protein 2; PDAC, pancreatic ductal adenocarcinoma; EV, empty vector. *P < 0.05, **P < 0.01.

We performed a CCK-8 assay to explore the effect of OSBP2 on PDAC cell proliferation. Our data showed that the OD value of si-OSBP2-infected ASPC-1 cells was lower than that of control ASPC-1 cells at different time points (*P <0.05*; **Figure 4A**), whereas the OD value of OSBP2-overexpressing BXPC-3 cells was higher than that of BXPC-3 cells at different time points (*P <0.05*; **Figure 4B**). This result indicated that OSBP2 inhibition suppressed PDAC cell proliferation in ASPC-1 cells and that OSBP2 overexpression promoted PDAC cell proliferation in BXPC-3 cells.

### OSBP2 Increases the Resistance of PDAC Cells to 5-FU and GEM

**Figure 5:**
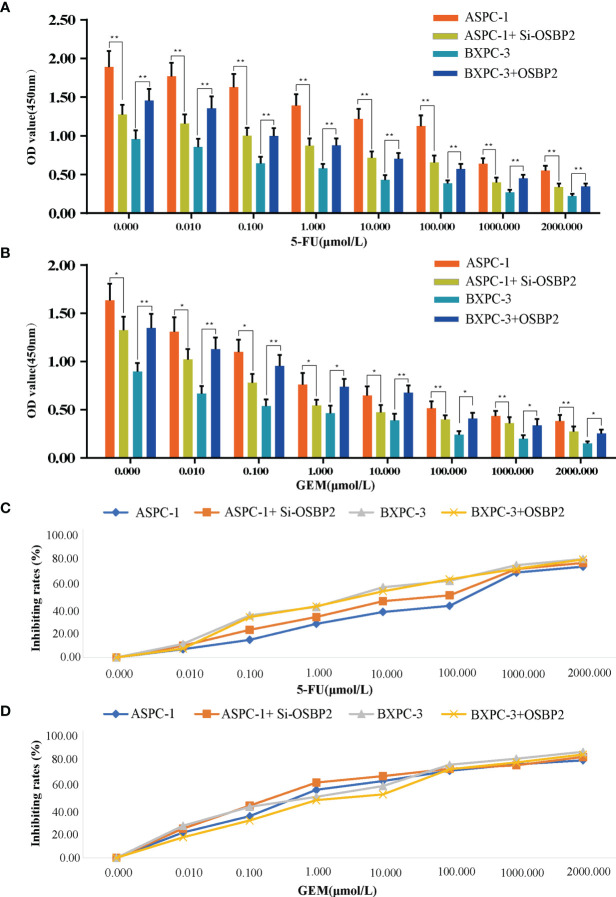
Effect of OSBP2 on the resistance of PDAC cells to 5-FU and GEM. **(A)** Low OSBP2 expression decreased the resistance of ASPC-1 cells to 5-FU (P < 0.01), and OSBP2 overexpression increased the resistance of BXPC-3 cells to 5-FU (P < 0.01). **(B)** Low OSBP2 expression decreased the resistance of ASPC-1 cells to GEM (P < 0.01), and OSBP2 overexpression increased the resistance of BXPC-3 cells to GEM (P < 0.01). **(C, D)** Increased concentrations of 5-Fu and GEM increased the inhibition of ASPC-1 and BXPC-3 cell lines. OSBP2, oxysterol-binding protein 2; 5-FU, 5-fluorouracil; GEM, gemcitabine. *P < 0.05, **P < 0.01.

Different cell lines (ASPC-1 cells, OSBP2-knockdown ASPC-1 cells, BXPC-3 cells, OSBP2-overexpressing BXPC-3 cells) were exposed to varying concentrations (0.000, 0.010, 0.100, 1.000, 10.000, 100.000, 1000.000, and 2000.000 μmol/l) of 5-FU and GEM for 72 h, and the OD values were examined. Our data showed that OSBP2 inhibition decreased the resistance of ASPC-1 cells to 5-FU and GEM, and OSBP2 overexpression increased the resistance of BXPC-3 cells to 5-FU and GEM (**Figures 5A, B**). As the drug concentration increased, the rate of growth inhibition increased (**Figures 5C, D**).

### OSBP2 Promotes the Proliferation of PDAC Cells *In Vivo*

**Figure 6:**
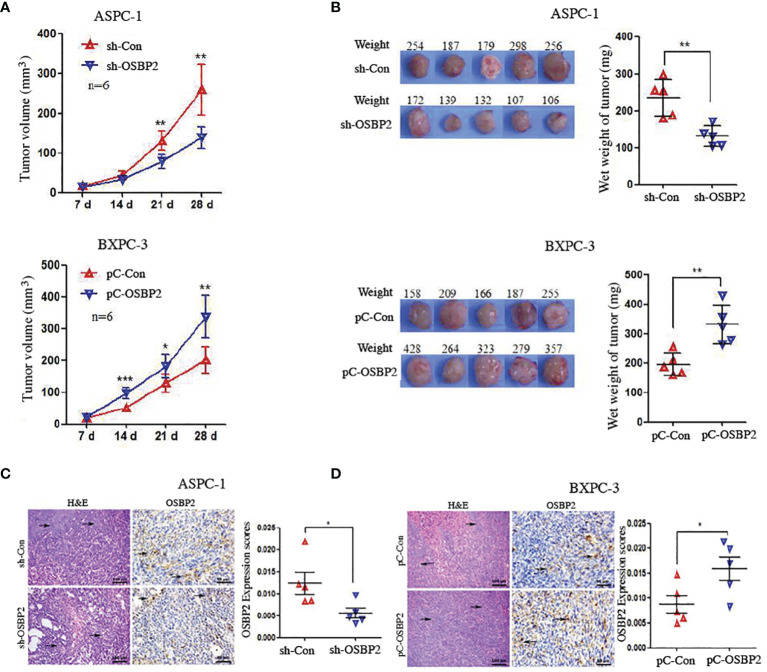
OSBP2 promotes the proliferation of PDAC cells *in vivo.* **(A)** OSBP2 knockdown decreased the tumor formation capacity of ASPC-1 cells, and OSBP2 overexpression increased the tumor formation capacity of BXPC-3 cells, as observed in xenograft models. **(B)** Photographs of excised tumors from nude mice and the tumor weights calculated at the end of the experiment are shown in the right panel. **(C, D)** Pathologic analysis of tissue sections from recipient mice at 4 weeks post-injection. H&E (magnification, 200×) and OSBP2 staining (magnification, 200×) were performed. Normalization of OSBP2 expression is shown on the right. OSBP2, oxysterol-binding protein 2; PDAC, pancreatic ductal adenocarcinoma; sh, shRNA; pC, pcDNA3.1 plasmid. *P < 0.05, **P < 0.01.

We examined the effect of OSBP2 on tumor growth using transplanted tumor mouse models. To this end, ASPC-1 cells, OSBP2-knockdown ASPC-1 cells, BXPC-3 cells, or OSBP2-overexpressing BXPC-3 cells were subcutaneously injected into the right posterior backs of BALB/c nude mice. Four weeks after tumor formation, we removed the tumor nodules and assessed their growth. We observed that OSBP2 inhibition suppressed the growth of ASPC-1-engrafted tumors and that OSBP2 overexpression accelerated the growth of BXPC-3-engrafted tumors (**Figure 6A**). In agreement with the tumor volumes, the weight of tumors derived from OSBP2-knockdown ASPC-1 cells was significantly lower than that of tumors derived from control ASPC-1 cells, and the weight of tumors derived from OSBP2-overexpression BXPC-3 cells was significantly higher than that of tumors the control (*P <0.05*; **Figure 6B**). Moreover, we performed H&E and OSBP2 staining of randomly selected mouse tumors. We observed that OSBP2 inhibition resulted in the downregulation of OSBP2 expression and that OSBP2 promotion resulted in the upregulation of OSBP2 expression (**Figures 6C, D**).

### OSBP2 Promotes the Metastasis of PDAC Cells *In Vivo*

**Figure 7:**
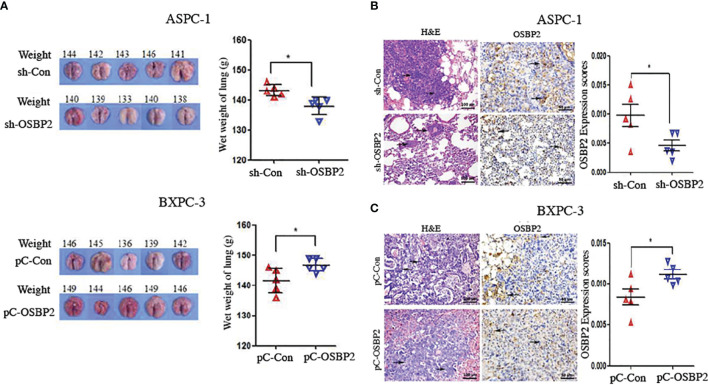
OSBP2 promotes PDAC cell invasion *in vivo*. **(A)** Photographs of excised tumors from SCID mice and the lung weights calculated at the end of the experiment. **(B, C)** Pathologic analysis of tissue sections from recipient mice at 8 weeks post-injection. H&E (magnification, 200×) and OSBP2 staining (magnification, 200×) were performed. Normalization of OSBP2 expression is shown on the right. OSBP2, oxysterol-binding protein 2; PDAC, pancreatic ductal adenocarcinoma; sh, shRNA; pC, pcDNA3.1 plasmid. *P < 0.05.

We conducted an *in vivo* metastasis assay by injecting OSBP2-knockdown ASPC-1 cells, ASPC-1 cells, BXPC-3 cells, and OSBP2-overexpressing BXPC-3 cells *via* tail vein into NOD/SCID mice. Eight weeks later, the animals were sacrificed, and metastasis progression was monitored. Both lungs were removed for histologic analysis, which revealed that the wet weight of both lungs of mice injected with OSBP2-knockdown ASPC-1 cells was lower than that of mice injected with ASPC-1 cells (*P <0.05*; **Figure 7A**). The wet weight of both lungs of mice injected with OSBP2-overexpressing BXPC-3 cells was heavier than that of mice injected with BXPC-3 cells (*P <0.05*; **Figure 7A**). H&E and OSBP2 staining of randomly selected mouse lung tissues showed that OSBP2 knockdown resulted in lower OSBP2 expression and that OSBP2 overexpression resulted in higher OSBP2 expression in the lung tissues compared to their respective controls (**Figures 7B, C**).

### OSBP2 Promotes EMT in PDAC Cells

**Figure 8:**
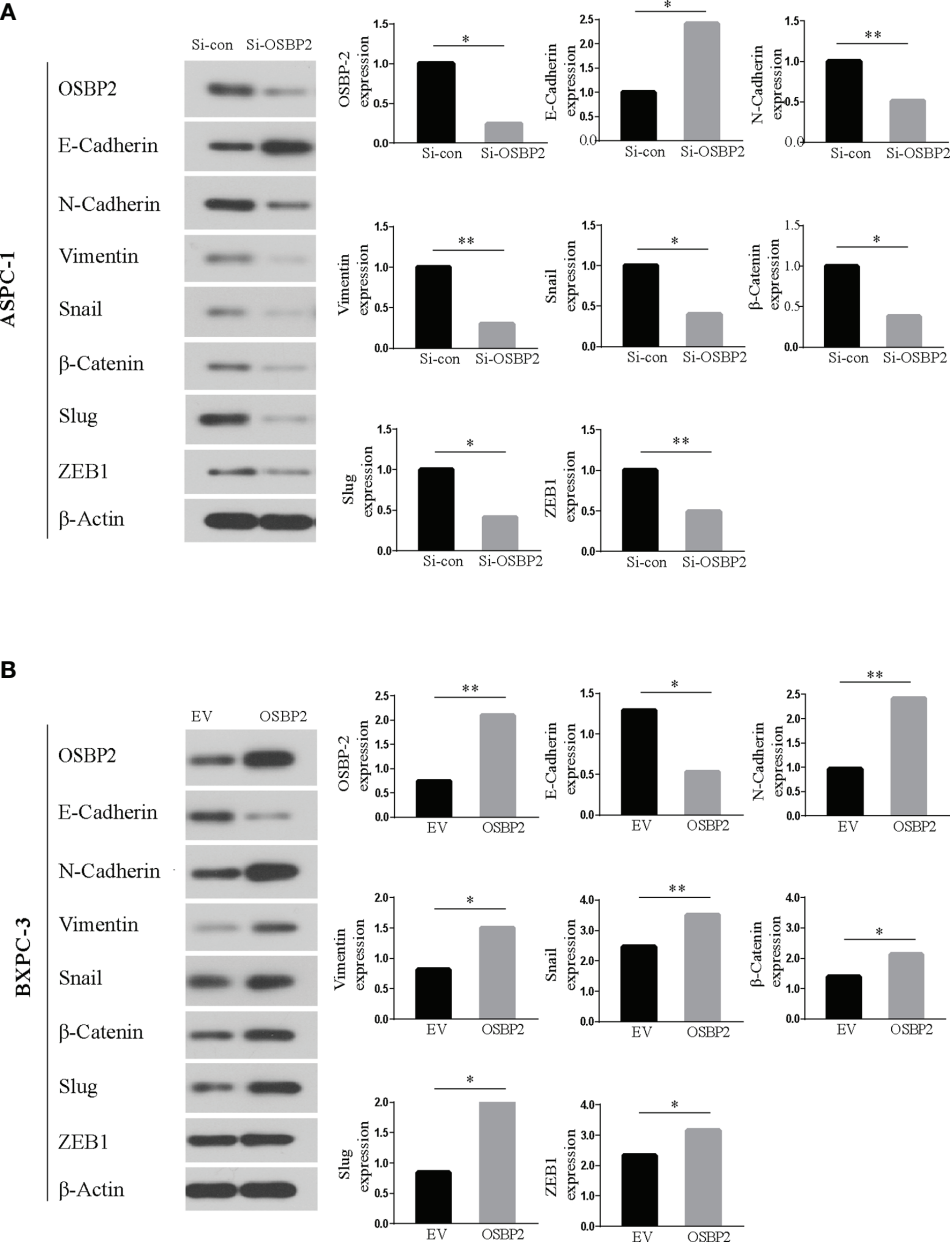
Western blot. **(A)** Western blot analysis revealed upregulated E-cadherin and downregulated N-cadherin, vimentin, and Snail expression in OSBP2-knockdown ASPC-1 cells. **(B)** Western blot analysis revealed upregulated N-cadherin, vimentin, and Snail and downregulated E-cadherin expression following overexpression of OSBP2 in BXPC-3 cells. OSBP2, Oxysterol-binding protein 2. *P < 0.05, **P < 0.01.

Western blot analysis showed that OSBP2 inhibition led to an increase in E-cadherin expression and a reduction in N-cadherin, vimentin, Snail, β-catenin, Slug, and ZEB1 expression in ASPC-1 cells (**Figure 8A**). By contrast, OSBP2 overexpression resulted in a reduction in E-cadherin expression and an increase in N-cadherin, vimentin, Snail, β-catenin, Slug, and ZEB1 expression in BXPC-3 cells (**Figure 8B**). The above results indicated that OSBP2 overexpression promoted the EMT process in PDAC cells.

## Discussion

At present, therapies for managing pancreatic cancer in patients are largely ineffective. PDAC generally predicts a poor prognosis, in part because the symptoms often do not appear obviously until the disease is too advanced for surgical treatment (2, 29, 30). The major reason for the extremely high mortality rate of PDAC is the invasive and metastatic phenotype of the cancer cells (31), therefore, early detection and intervention remain the greatest challenges in the treatment of this malignancy. Understanding the molecular pathogenesis of PDAC is a mainstream focus of cancer research. In this study, we first demonstrated that OSBP2 could positively promote PDAC cell migration and invasion *in vitro* and *in vivo* and that this potentially occurs *via* EMT.

The domains of OSBPs and ORPs are conserved, namely, high affinity lipid domains that are apt to bind to sterols. These domains of OSBP and ORPs together with the domains of PH and FFAT participate in sterol/lipid transfer and signal transduction between organelle membranes (32). Diffusion and metastasis are characteristics of malignant tumors. The levels of various OSBPs in tumor cells are different from those in normal cells at the mRNA or protein level, indicating the important role of these proteins in tumorigenesis. ORP inhibitors (named ORPphilins), acting as antiproliferative agents, are crucial for the inhibition of tumor growth and are capable of attenuating OSBP or ORP4L (28, 33). The effects of OSBPs on tumor migration and invasion in various cancer types were revealed by previous studies (34, 35). However, there were no data in the literature describing OSBP2 expression and function in pancreatic cancer. In our study, we initially found that OSBP2 was expressed in not only PDAC cell lines but also primary PDAC tissues. The expression of OSBP2 in PDAC tissues was obviously higher than that in paracarcinoma tissues; more importantly, patients with high OSBP2 expression had a worse prognosis, which suggests that OSBP2 could serve as a potential tumor marker for PDAC patients.

PDAC has the lowest 5-year survival rate (8%) among all tumors, and this rate dwindles to 3% among individuals initially diagnosed with terminal disease (36). Surgery has an efficacious therapeutic value in this type of cancer, but only 20% of patients are eligible (37, 38). GEM is still the first-line chemotherapeutic for patients who are not eligible for radical resection, and 5-FU is also used as an alternative to GEM, but chemotherapy has limited efficacy for treating metastatic PDAC (39, 40). We first demonstrated that OSBP2 could obviously affect the chemosensitivity of PDAC in patients. When OSBP2 was specifically knocked down, the apoptotic rates of PDAC cells treated with GEM and 5-FU significantly increased. Some research shows that there exists a connection between OSBP family members and malignant tumor cell proliferation and metastasis. Our *in vitro* and *in vivo* experiments revealed that OSBP2 not only promotes PDAC cell proliferation but also enhances invasion and metastasis. The abovementioned preliminary results imply that OSBP2 plays a key role in neoplastic progression.

To improve the dismal survival and prognosis of PDAC patients, new effective therapeutic strategies are urgently required. Previous studies have revealed that EMT is responsible for early-stage tumor cell dissemination and is positively involved in the invasion and metastasis of PDAC (13, 41, 42). Previous work indicated that members of the OSBP family could regulate the subcellular distribution of ORP–VAPA complexes and their impacts on organelle structure and consequently affect malignant tumor cell invasion and metastasis, potentially *via* changes in vimentin distribution (43). Our research explored the effect of OSBP2 on the phenotypic transformation of PDAC cells by knocking down and overexpressing OSBP2. We demonstrated for the first time that OSBP2 knockdown and overexpression could significantly change the expression of EMT-related markers in PDAC cells. When OSBP2 was knocked down, the levels of the mesenchymal markers N-cadherin and Vimentin and the EMT transcription factors (TFs) β-catenin, ZEB1, Slug, and Snail were obviously decreased (44). Therefore, we hypothesized that OSBP2 overexpression could be recognized as a potential inducer of EMT.

Diseases with a bleak prognosis urgently need novel therapeutic programs. However, the understanding of tumor biology and taxonomy with regard to PDAC is far from fully elucidated, which can hinder improvements of the treatment effects of available therapies. Here, we provided relevant evidence indicating that OSBP2 is a hallmark of malignancy, offered strong data showing that it regulates EMT through novel mechanisms and highlighted its potential as a marker for stratifying pancreatic cancer patient.

## Data Availability Statement

The datasets presented in this study can be found in online repositories. The names of the repository/repositories and accession number(s) can be found in the article/supplementary material.

## Ethics Statement

The studies involving human participants were reviewed and approved by The Ethics Committee of Scientific Research and Clinical Experiment of the First Affiliated Hospital of Zhengzhou University. The patients/participants provided their written informed consent to participate in this study. The animal study was reviewed and approved by The Ethics Committee of First Affiliated Hospital of Zhengzhou University.

## Author Contributions

SH performed experiments, contributed to the analysis and interpretation of the data and was a major contributor in writing the manuscript. XZ and KL performed the experiments and contributed to drafting and revising the manuscript. LJ contributed to the data analysis and revision of the manuscript. RL and JJ contributed to the conception and design of the study, the data analysis and interpretation, and the writing and revision of the manuscript. All authors read and approved the manuscript and agree to be accountable for all aspects of the research in ensuring that the accuracy or integrity of any part of the work are appropriately investigated and resolved.

## Funding

The present study was supported by The Key Scientific Research Project Plan of Henan University (grant no. 20A320037).

## Conflict of Interest

The authors declare that the research was conducted in the absence of any commercial or financial relationships that could be construed as a potential conflict of interest.

## Publisher’s Note

All claims expressed in this article are solely those of the authors and do not necessarily represent those of their affiliated organizations, or those of the publisher, the editors and the reviewers. Any product that may be evaluated in this article, or claim that may be made by its manufacturer, is not guaranteed or endorsed by the publisher.
